# Flexible Dual‐Modal Sensors Based on Single‐Crystalline Silicon Membranes for Continuous Monitoring of Photoplethysmography and Skin Temperature

**DOI:** 10.1002/advs.202506348

**Published:** 2025-06-25

**Authors:** Yanle He, Haonan Zhao, Xiaozhong Wu, Junjie Zhou, Ailing Yin, Deyu Meng, Wenli Sun, Qinglei Guo

**Affiliations:** ^1^ School of Integrated Circuits Shandong University Jinan 250100 P. R. China; ^2^ State Key Laboratory of Materials for Integrated Circuits Shanghai Institute of Microsystem and Information Technology Chinese Academy of Sciences Shanghai 200050 P. R. China

**Keywords:** Silicon membranes, epidermal electronics, flexible dual‐modal sensors, decoupling mechanism, health monitoring

## Abstract

Flexible dual‐modal sensors that can monitor two signals play important roles in biomedical applications, along with the recent progresses of epidermal or bioimplantable electronic devices with diagnostic or therapeutic functionalities. However, techniques for flexible dual‐modal sensors mainly rely on integrating multiple sensing units on a platform, thus inducing extra costs and complexities associated with the fabrication and data processing. In this paper, a flexible dual‐modal sensor is presented, which contains only one sensing unit, i.e., single‐crystalline silicon‐based diode, for the real‐time, independent, and continuous monitoring of light and temperature. Operation modes for photodetection and temperature sensing of the flexible dual‐modal sensor are switchable by controlling the biased condition. In addition, both experimental results and theoretical calculations indicate that the crosstalk between light and temperature can be conveniently decoupled by short‐circuit current and forward current of silicon‐based diodes. Finally, the flexible dual‐modal sensor is implemented as a platform to realize the continuous monitoring of photoplethysmography (PPG) and skin temperature of fingertips. These presented results offer paths to the construction of multifunctional, flexible bioelectronic devices or integrated platforms for biomedical uses.

## Graphical abstract

A flexible light‐temperature dual‐modal sensor is demonstrated with silicon‐based diode, of which the operation mode, i.e., photodetection and temperature sensing, can be conveniently controlled by the biased condition. Engineering demonstrations illustrate the applicability of the sensor to continuously monitor photoplethysmography and skin temperature of fingertips for biomedical applications.

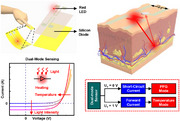

## 1 Introduction

Recent advances in material science and device architectures have led to the development of flexible dual‐modal sensors, which provide numerous opportunities for applications in biomedical engineering, such as health monitoring,^[^ ^1^, ^2^ ^]^ preventive medicine,^[^ ^3^ ^]^ disease diagnosis,^[^ ^4^, ^5^ ^]^ and many others.^[^ ^6^, ^7^, ^8^ ^]^ Compared to traditional flexible sensors, one of the most important advantages of flexible dual‐modal sensors reflects in their capabilities to provide more information associated with the health status,^[^ ^9^ ^]^ because they can monitor two physiological signals without crosstalk.^[^ ^10^ ^]^ Currently, technical routes for the fabrication of flexible dual‐modal sensors mostly rely on integrating multiple units that have different sensing functionalities on a platform.^[^ ^11^ ^]^ Although monitoring of each signals can be realized by independent operation of the corresponding sensor, compensating circuits or components are normally required to eliminate the crosstalk.^[^ ^12^ ^]^ Besides, high complexities and costs resulted from the integration of different sensing units or components, along with the limited integration scale, may also hinder their further development. These problems can be circumvented, to some extent, with flexible dual‐modal sensors that have only one sensing unit.^[^ ^13^ ^]^ In these sensors, external stimuli can be transduced into two independent electrical signals, thus permitting their capabilities of dual‐modal sensing. With the rapid development of materials science and device fabrication technology, various flexible dual‐modal sensors, such as pressure‐temperature,^[^ ^14^, ^15^, ^16^ ^]^ strain‐temperature,^[^ ^17^, ^18^, ^19^ ^]^ and strain‐pressure,^[^ ^20^, ^21^, ^22^ ^]^ have been widely developed. However, flexible dual‐modal sensors that contain one sensing unit to detect light and temperature have been rarely reported, particularly in field of biomedical engineering, possibly due to light, unlike temperature, is not a typical physiological signal.

Nevertheless, light as an effective medium can provide specific localization and stimulation to cells or cell populations, thus being of great importance to reveal the physiological behavior of living organisms. For instance, PPG sensors that can detect the pulsation of blood in the arteries, which strongly affects the absorption of the incident light, can be utilized to access the cardiac functions through the continuous monitoring of pulse,^[^ ^23^, ^24^ ^]^ blood oxygen levels,^[^ ^25^, ^26^ ^]^ or other related hemodynamic parameters. The photoelectric conversion can build‐up the interaction between optoelectronic devices and neural signals,^[^ ^27^ ^]^ thus providing optogenetic modulation,^[^ ^28^, ^29^ ^]^ wireless optoelectronic stimulation,^[^ ^30^, ^31^ ^]^ and optoelectronic sensing to cells, tissues, or living systems,^[^ ^32^, ^33^ ^]^ which is of great significance to both fundamental researches of neurosciences and the diagnosis/treatment of neurological diseases. In these examples, additional health status information would be provided to facilitate precise diagnosis and/or therapeutic treatments of diseases if the physiological signal of temperature can be monitored. For example, Bai et al. develop a multifunctional optoelectronic platform,^[^ ^34^ ^]^ with which the cerebral oxygenation and cerebral temperature of a mice model can be monitored by the photodetection and temperature sensing components, respectively, thus strongly supporting the precise evaluation of its physiological status and neural activity. Therefore, the development of flexible light‐temperature dual‐modal sensor is of great significance, particularly for biomedical uses.

In terms of material strategies for the fabrication of flexible light‐temperature dual‐modal sensor, organic or 2D semiconductors could offer excellent mechanical flexibility,^[^ ^35^, ^36^ ^]^ however, they involve one or more disadvantages in poor electrical performance and stability, low thermal durability, high cost of fabrication, and/or lack of uniformity. Although oxide semiconductors enable a simple and low‐temperature manufacturing process, they suffer from the poor and susceptible electrical properties.^[^ ^37^ ^]^ Among various sensing materials, silicon is of interest for the fabrication of high‐performance sensors due to its good and long‐term stable electrical and optical properties.^[^ ^38^ ^]^ Besides, silicon is naturally compatible with the state‐of‐the‐art semiconductor technology, thus providing possibilities for the convenient and mass production of sensors. Although silicon is normally known as a rigid and brittle materials, the bending stiffness can be drastically reduced as the thickness reduces. As a result, silicon nanomembrane (SiNM) is particularly suitable for the fabrication of flexible electronics.^[^ ^39^ ^]^ Nevertheless, utilizing SiNMs to fabricate flexible light‐temperature dual‐modal sensor is challenged by decoupling the crosstalk between light and temperature, which is rarely investigated.

In this work, we report material strategies, devices, and working principles of a flexible light‐temprature dual‐modal sensor, containing only one sensing unit. Single‐crystalline silicon membrane‐based diode, of which the manufacturing process is naturally compatible with the state‐of‐the‐art semiconductor technology, serves as the key sensing component. Systematic experimental and theoretical investigations reveal that the operation modes of silicon‐based diodes for photodetection and temperature sensing is switchable by its biased condition. The illuminated light and temperature can be transduced into independent eletrical signals, i.e., short‐circuit current and forward current, respectively, thus enabling the decoupling of the corresponding crosstalk. As an example for biomedical uses, continuous monitoring of PPG and skin temperature of fingertips are demonstrated by the flexible dual‐modal sensor. Integrating such silicon‐based flexible dual‐modal sensor with other bioelectronic devices or components into a multifunctional flexible platform should provide numerous opportunities for fundamental studies and potential applications in biomedical engineering.

## 2 Results and Discussion

**Figure 1:**
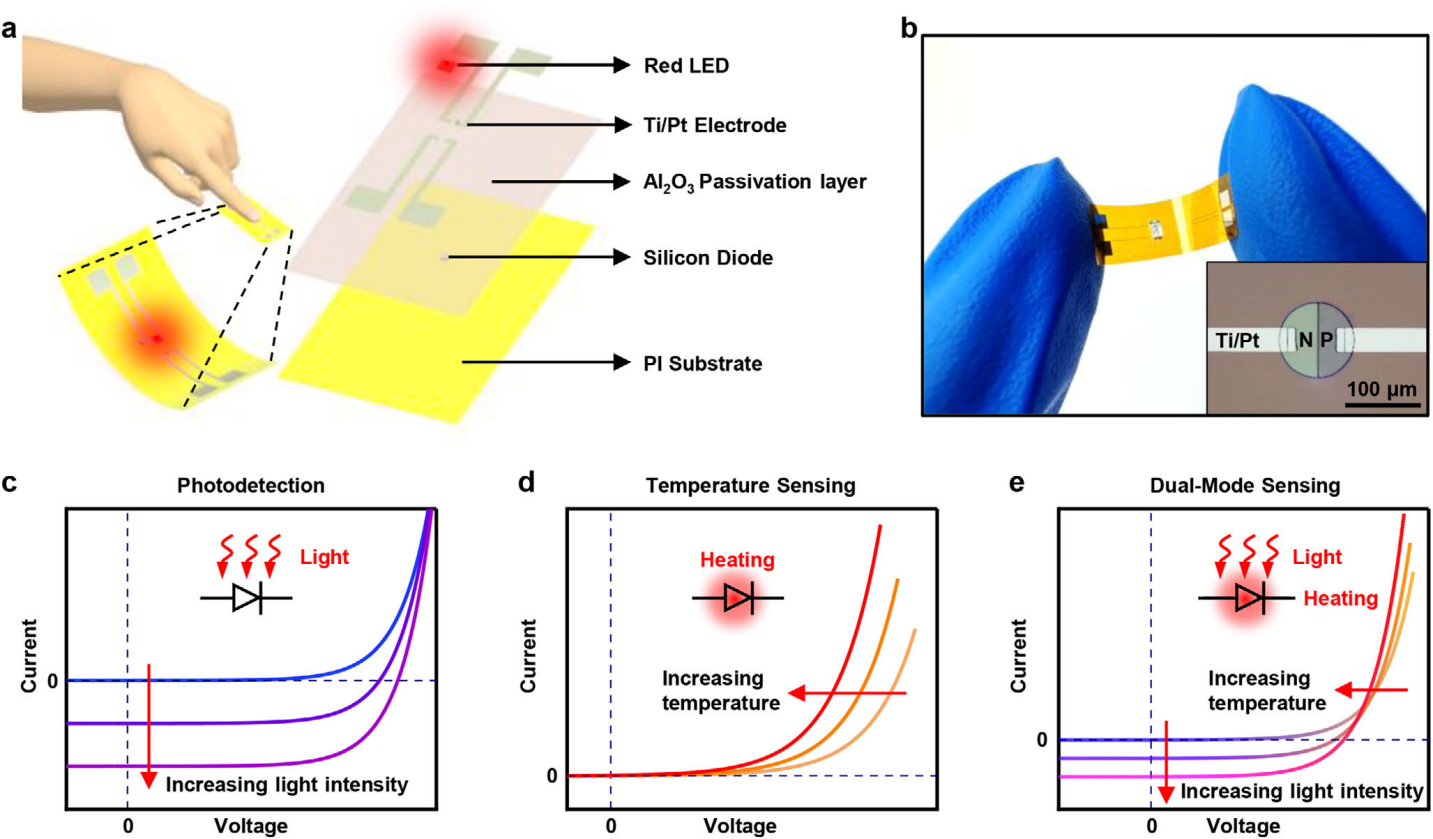
a) Exploded schematic of key materials, components, and architectures of the flexible dual‐mode sensor. b) Optical image of the sensor in a bending status. The inset is the optical microscope image of a silicon‐based PN junction. Schematic plots of the current‐voltage characteristics of the proposed flexible dual‐mode sensor operating at photodetection mode c), temperature sensing mode d), and dual‐mode sensing of light and temperature e). The blue dotted lines in c) and e) represent the coordinate axis that crosses the zero point.

The schematic diagram in **Figure** **1**a illustrates key materials and structures of the proposed flexible dual‐modal sensor, which consists of a commercial light emitting diode (LED), Ti/Pt electrodes, alumina passivation layer, a silicon‐based diode, and the flexible polyimide (PI) substrate from top to bottom. As human fingers contact with the flexible sensor, the skin temperature and pulse can be determined by the measured current‐voltage characteristics of silicon‐based diodes.^[^ ^40^ ^]^ The optical image in Figure 1b demonstrates good flexibility of the fabricated dual‐modal sensor, and a circular silicon‐based diode with a diameter of 100 µm appears in the inset. Figure S1 (Supporting Information) schematically illustrates the fabrication process of flexible silicon‐based diodes, in which the transfer printing technique is utilized to transfer the released silicon membrane onto flexible PI substrate. The alignment accuracy and the device yield associated with the transfer printing process used for fabricating a 6×6 flexible silicon‐based diode array can be well controlled, as shown in Figure S2 (Supporting Information). As a result, ≈86% of the diode array have the I_on_/I_off_ (at ±1 V) of 10^6^, and 80% of them have a turn‐on voltage of 0.7 V.

In the proposed flexible dual‐modal sensor, the silicon‐based diode serves as the key component for the sensing of both light and temperature. For the light sensing, the photocurrent of a diode at the reverse biased region will monotonously increase with the increase of the illuminated light, as schematically shown in Figure 1c. As a result, the power density of the illuminated light can be extracted by the measured photocurrent. Notably, the photocurrent at the forward‐biased region is almost unchanged because of the dominant contribution in forward bias current by the diffusion of the majority carriers.^[^ ^41^ ^]^ On the other hand, the current of the diode operating at the forward region (with the constant voltage) significantly increases with the increase of the temperature, as displayed in Figure 1d. Therefore, the fabricated flexible Si‐based diode can be used for the monitoring of the skin‐temperature. The reverse saturation current, in this case, exhibits negligible change. In other words, the photodetection mode and temperature sensing mode can be independently operated for diodes, which is switchable by the biased condition, thus building‐up the fundamentals for the dual‐modal sensing of both light and temperature, as illustrated in Figure 1e. Details about the corresponding decoupling mechanism will be discussed later.

**Figure 2:**
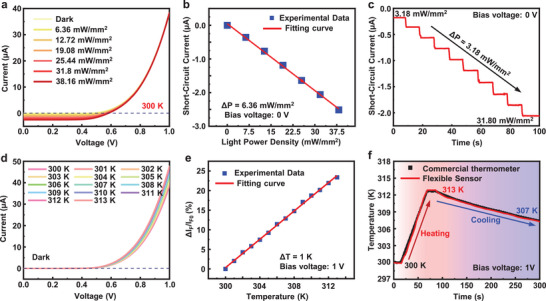
a) Current‐voltage characteristics of the sensor operating at the photodetection mode. A 635 nm laser with tunable light power densities is used as the illuminated light source. The temperature is set as 300 K. b) Experimental (blue dots) and fitting (red line) results of the short‐circuit current extracted at 0 V varying with the light power density. c) Real‐time variations in the short‐circuit current obtained from the sensor, which is continuously illuminated by the laser with stepped rising light power densities. d) Current‐voltage characteristics of the sensor in the dark at different temperatures. e) Experimental (blue dots) and fitting (red line) results of the percentage variations in forward current extracted at 1 V of the sensor at different temperatures. f) Real‐time monitoring of temperature variations obtained from the fabricated sensor (red curve) and a commercial thermometer (black dots). The blue dotted line in both a) and d) represents the horizontal axis that crosses the zero point.

To evaluate the photodetection characteristics of the flexible dual‐modal sensor, an experimental apparatus consisting of a custom‐made optoelectronic testing platform, a light power meter, and a 635 nm laser is constructed. **Figure** **2**a shows the measured current‐voltage characteristics of the sensor illuminated with linearly increased power densities with an interval of 6.36 mW mm^−2^. As the light power density increases, the photocurrent increases accordingly, as shown in Figure S3 (Supporting Information). Moreover, due to the photovoltaic effect of the SiM‐based diode, the short‐circuit current (biased at 0 V) increase linearly with the illuminated light power density, as summarized in Figure 2b, suggesting the potential for the use of self‐powered photodetection.^[^ ^42^, ^43^, ^44^, ^45^ ^]^ Figure 2c shows the short‐circuit current measured from the flexible dual‐modal sensor, which is illuminated with steadily increased and stepped power densities (power density interval: 3.18 mW mm^−2^). The obtained short‐circuit current maintains a consistent value once the light power density is constant, suggesting the stable optoelectronic response of the fabricated flexible dual‐modal sensor. As shown in Figure S4 (Supporting Information), current‐voltage characteristics of the sensor remain stable under different degrees of bending and mechanical cycling tests, demonstrating its good flexibility. Long‐term stable photodetection is demonstrated by a pulsed light illumination with the switching frequency and power density of 1 Hz and 0.13 mW mm^−2^, respectively, as revealed in Figure S5 (Supporting Information). Under other illumination conditions, the sensor also exhibits stable and linear optoelectronic responses, as shown in Figure S6 (Supporting Information). The demonstrated stable photodetection lays down the foundation for applications in wearable optical systems.^[^ ^46^, ^47^ ^]^ Figure S7 (Supporting Information) shows the short‐circuit current varying with a one‐cycle light illumination, and both the rising time (t_on_) and the falling time (t_off_) of the sensor are ≈40 ms, which is sufficient for most of biomedical applications, such as the monitoring of heart rate, tissue oxygenation, and neuronal dynamics.^[^ ^48^, ^49^, ^50^ ^]^ Particularly, the monitoring of PPG under different physiological conditions is also available, which will be discussed later.

In addition to the photodetection, the fabricated flexible dual‐modal sensor can be also utilized for temperature sensing, because of the temperature‐dependent current‐voltage characteristics of Si‐based diodes. Figure 2d shows the current‐voltage characteristics of the sensor at different temperatures ranging from 300 K to 313 K with an interval of 1 K. Notably, variations in temperature significantly affect the forward current especially the diode is turned‐on. The enlarged results in the bias voltage range of 0.99–1 V, as shown in Figure S8 (Supporting Information), clearly reveal this tendency. Figure 2e shows the percentage change in forward current at the bias voltage of 1 V varying with the temperature, which exhibits an obvious linear relationship. As a result, the temperature coefficient of current (TCC) is calculated as 1.79%/°C, with the value being much higher than most of previously reported Si‐based temperature sensors, as shown in Table S1 (Supporting Information). The good repeatability of the sensor is demonstrated by its highly consistent forward current (bias voltage: 1 V) at seven cycles of heating (313 K) and cooling (300 K) treatments, as shown in Figure S9 (Supporting Information). Moreover, the real‐time temperature variation of a hot plate during heating and cooling operations can be accurately monitored by the sensor, which is coincident with a commercial sensor (Figure 2f). Besides, a small resolution of 0.1 K is evidenced, as shown in Figure S10 (Supporting Information), suggesting great potentials for applications in the monitoring of skin (or other organs) temperature. Details about the comparison on key parameters of the dual‐modal sensors with previously reported works appear in Table S2 (Supporting Information).

**Figure 3:**
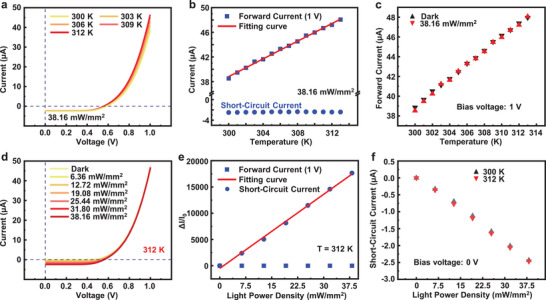
a) Current‐voltage characteristics of the sensor illuminated with a constant light power density of 38.16 mW mm^−2^ at different temperatures. b) Variations of forward current (blue square dots, biased voltage: 1 V) and short‐circuit current (blue circular dots) of the sensor with temperature. The illuminated light power density is maintained as 38.16 mW mm^−2^. c) Forward current of the fabricated flexible silicon‐based diode varying with the temperature with (red dots) and without (black dots) the light illumination. d) Current‐voltage characteristics of the sensor illuminated with different light power densities, and the temperature is maintained at 312 K. e) Relative change of forward current (blue square dots, biased voltage: 1 V) and short‐circuit current (blue circular dots) of the sensor varying with light power density, and the temperature is constant as 312 K. f) Short‐circuit current of the fabricated flexible silicon‐based diode varying with the light power density at different temperatures. The blue dotted lines in a) and b) represents the coordinate axis that crosses the zero point.

It should be noted that the above characterizations on either photodetection or temperature sensing of the fabricated sensors are performed independently. For a light‐temperature dual‐mode sensor, the ability of decoupling the crosstalk between light and temperature is essential. Therefore, the photodetection performances of the sensor at different temperatures, as well as the temperature sensing performances under different light illumination conditions, are evaluated. **Figure** **3**a shows the current‐voltage characteristics of the sensor under the light illumination when the applied temperature gradually rises with an interval of 3 K. The short‐circuit current maintains constant, and the forward current gradually increases. In other words, the short‐circuit current is exclusively determined by the illuminated light power density, but not the temperature, as confirmed in Figure S11 (Supporting Information). These observations can be further confirmed by the summarized results about the changes of both forward current (biased at 1 V) and short‐circuit current with the applied temperature (Figure 3b). Moreover, the temperature sensing performances of the sensor has negligible change at dark or light illumination conditions, as shown in Figure 3c.

Furthermore, the effect of light illumination on the temperature sensing performance is estimated. When the sensor is illuminated by a laser (635 nm in wavelength) with gradually increased light power densities at an interval of 6.36 mW mm^−2^, as shown in Figure 3d, the obtained forward current has unnoticeable change but is exclusively governed by the applied temperature, and the short‐circuit current gradually increases. In other words, the forward current is exclusively determined by the applied temperature, but not the illuminated light power density, as confirmed in Figure S12 (Supporting Information). Figure 3e summarizes the relative changes of the forward current (biased at 1 V) and short‐circuit current varying with the light power density. And the applied temperature is constant as 312 K. Moreover, the photodetection performances of the sensor has negligible change with the applied temperature, as shown in Figure 3f. Notably, the operational limits for both temperature and light power density at which decoupling remains effective are identified as 60 °C and 33 mW mm^−2^, respectively, as shown in Figure S13 (Supporting Information) and Supplementary Note 1. These presented results provide reliable experimental demonstrations for the fabricated flexible dual‐modal sensor to decouple the crosstalk between light and temperature. The photodetection mode and temperature sensing mode of the sensor is switchable by the biased condition that variations in short‐circuit current and forward current can be utilized to determine the illuminated light and temperature, respectively.

**Figure 4:**
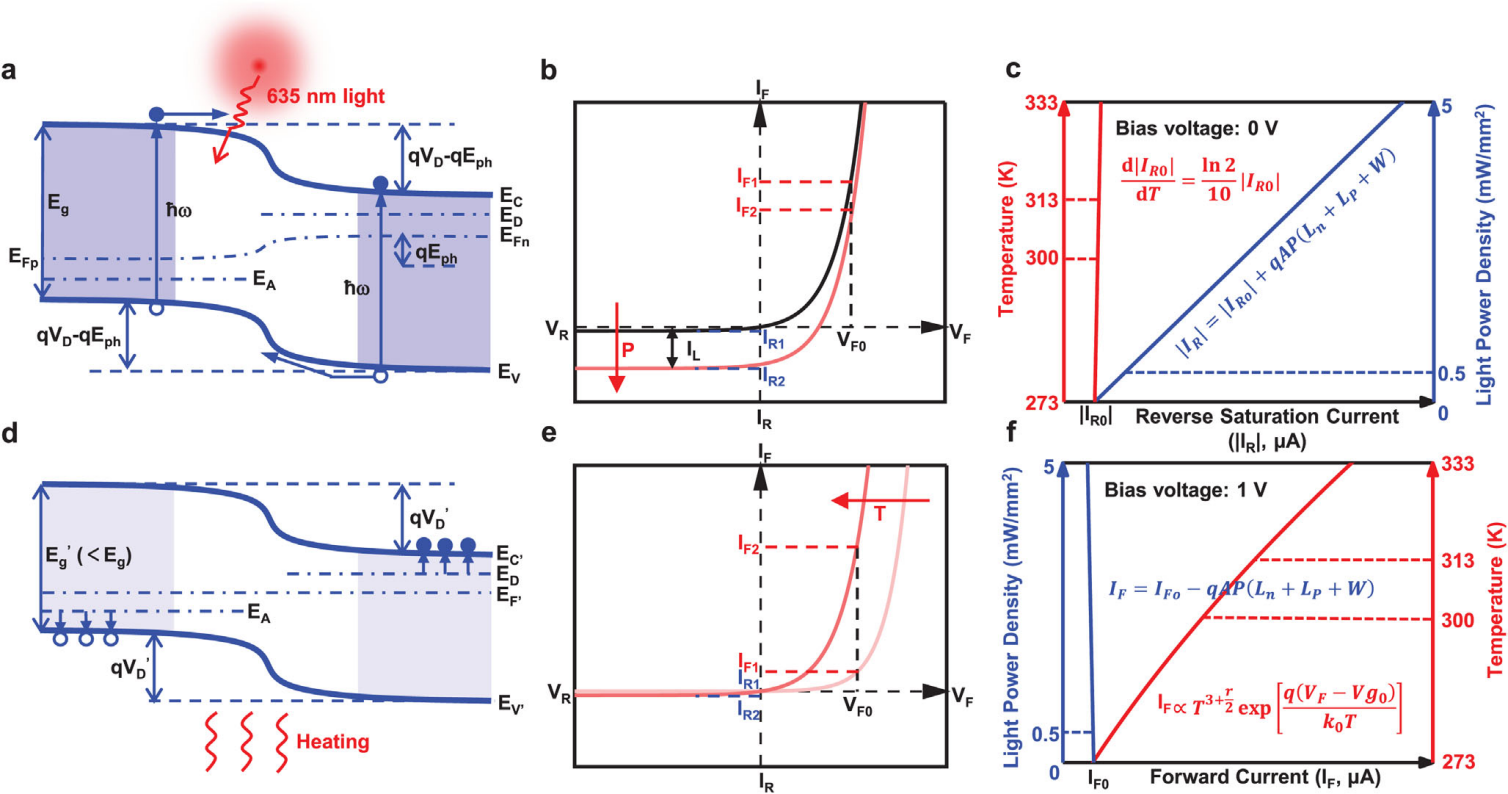
a) Energy band diagram of silicon photodiode under the 635 nm laser illumination. b) Schematic plots of the current‐voltage characteristics under different light illumination conditions. c) Theoretically calculated results about the short‐circuit current varying with temperature (red curve) and light power density (blue curve). The highlighted variation ranges of temperature (300‐313 K) and light power density (0–0.5 mW mm^−2^) denote the skin temperature variations and typical light power density required for PPG applications. d) Energy band diagram of silicon photodiode treated by the heating. e) Schematic plots of the current‐voltage characteristics with elevated heating temperature. f) Theoretically calculated results about the forward current (bias voltage: 1 V) varying with temperature (red curve) and light power density (blue curve).

Theoretical mechanism for decoupling the crosstalk between light and temperature of the fabricated flexible dual‐modal sensor starts with building‐up the energy band diagram of Si‐based diode in the equilibrium state (Figure S14, Supporting Information). When the diode is illuminated by a 635 nm light, the generated electron‐hole pairs on both sides move in reverse because of the built‐in potential in the depletion region, as illustrated in **Figure** **4**a, forming a photogenerated current, *I*_*L*_, flowing from the n‐region to the p‐region. The photovoltaic electromotive force, *E*_*p* *h*_, results in a decrease in the barrier height. For an ideal PN junction, the current‐voltage (I‐V) characteristics can be described by the Shockley equation,^[^ ^51^ ^]^

(1) $$I=I_{R0}\left[\exp\left(\frac{qV}{k_{0}T}\right)-1\right]$$

where *I*_*R0*_ is reverse saturation current, *k*_*0*_ is Boltzmann constant, *T* is temperature, and *q* is basic charge. Since the photogenerated current (*I_L_*) has an opposite direction with the current (*I*) of the PN junction, the corresponding I‐V characteristics under the illumination can be written by,^[^ ^52^ ^]^

(2) $$I=I_{R0}\left[\exp\left(\frac{qV}{k_{0}T}\right)-1\right]-I_{L}$$

where

(3) $$I_{L}=qAP\left(L_{n}+L_{P}+W\right)$$

With the light illumination, *I_L_* is mainly contributed by extra holes (*kL*_*p*_ *G*_*op*_) generated on the n‐region, extra electrons (*kL*_*n*_ *G*_*op*_) generated on the p‐region, and extra carriers (*kWG*_*op*_) generated within the depletion region. Herein, *k* is coefficient of direct proportion, *G*_*op*_ is photogeneration rate of carriers, and *L*_*n*_ and *L*_*p*_ are the diffusion lengths of minority carriers. In Equation (3), *A* is coefficient of direct proportion containing *k*, *P* is the light power density that is proportional to *G*_*op*_ in a certain range of light intensity, and *W* is the width of the depletion region. Based on the above analyses, the current‐voltage characteristics of Si‐based diode under dark and light conditions are plotted in Figure 4b.

Based on Equations (2) and (3), the reverse saturation current of Si‐based diode under the light illumination (*I_R_*) can be simplified as,

(4) $$\left[I_{R}\right]=\left|I_{R0}\right|+qAP\left(L_{n}+L_{P}+W\right)$$

considering exp[(*qV*)/(*k*_*0*_ *T*)] is ≈0 under the reverse biased condition. In this case, crosstalk induced by the temperature is mainly contributed by the former term of Equation (4), since the slight increase in *I_L_* with temperature due to a decrease in the band gap of silicon can be ignored.^[^ ^53^, ^54^ ^]^ For the reverse saturation current, *I*_*R0*_, it will double for every 10 K rise in temperature, and can be written as,

(5) $$\frac{dI_{R0}}{dT}=-\frac{\ln2}{10}I_{R0}$$

According to Equations (4) and (5), the reverse saturation currents of Si‐based diode varying with illuminated light (blue curve) and temperature (red curve) are sketched. As shown in Figure 4c, within the range of human skin temperature variations (e.g., 300–313 K), the induced change in *I*_*R*_ is far less than that resulted from the light illumination (even at a small light power density range: 0–0.5 mW mm^−2^). Therefore, the crosstalk induced by temperature during the photodetection mode is negligible.

When the applied temperature increases, both the band gap of silicon and the corresponding barrier height, *V*_*D*_, of the Si‐based diode decrease, as schematically presented in Figure 4d. Besides, the diffusion constant, diffusion length, and density of equilibrium carriers in silicon are all depended on the temperature. Therefore, temperature will strongly affect the I‐V characteristics of the Si‐based diode, as displayed in Figure 4e. Especially for the forward biased condition, the relationship between the forward current and temperature can be expressed as,^[^ ^40^ ^]^

(6) $$I_{F}\propto T^{3+\frac{\gamma }{2}}\exp\left(\frac{q\left(V_{F}-V_{g0}\right)}{k_{0}T}\right)$$

where *γ* is a constant, *V*_*g0*_ is the potential difference between bottom of conduction band and top of valence band at absolute zero Kelvin. Since *T* ^*(3+γ/2)*^ changes slowly with temperature, the latter item of Equation (6) will contribute mainly to the temperature‐dependent forward current. Based on Equations (2) and (6), the forward currents of Si‐based diode varying with illuminated light (blue curve) and temperature (red curve) are sketched. As shown in Figure 4f, the forward current significantly increases with the increasing temperature. On the other hand, slight variations in the forward current induced by the light illumination can be observed. For practical applications, e.g., PPG, most of commercially available sensors use LEDs with light power densities less than 0.5 mW mm^−2^, as summarized in Table S3 (Supporting Information). This small range of light power density further reduces the forward current variation, with negligible crosstalk, during the temperature sensing.

**Figure 5:**
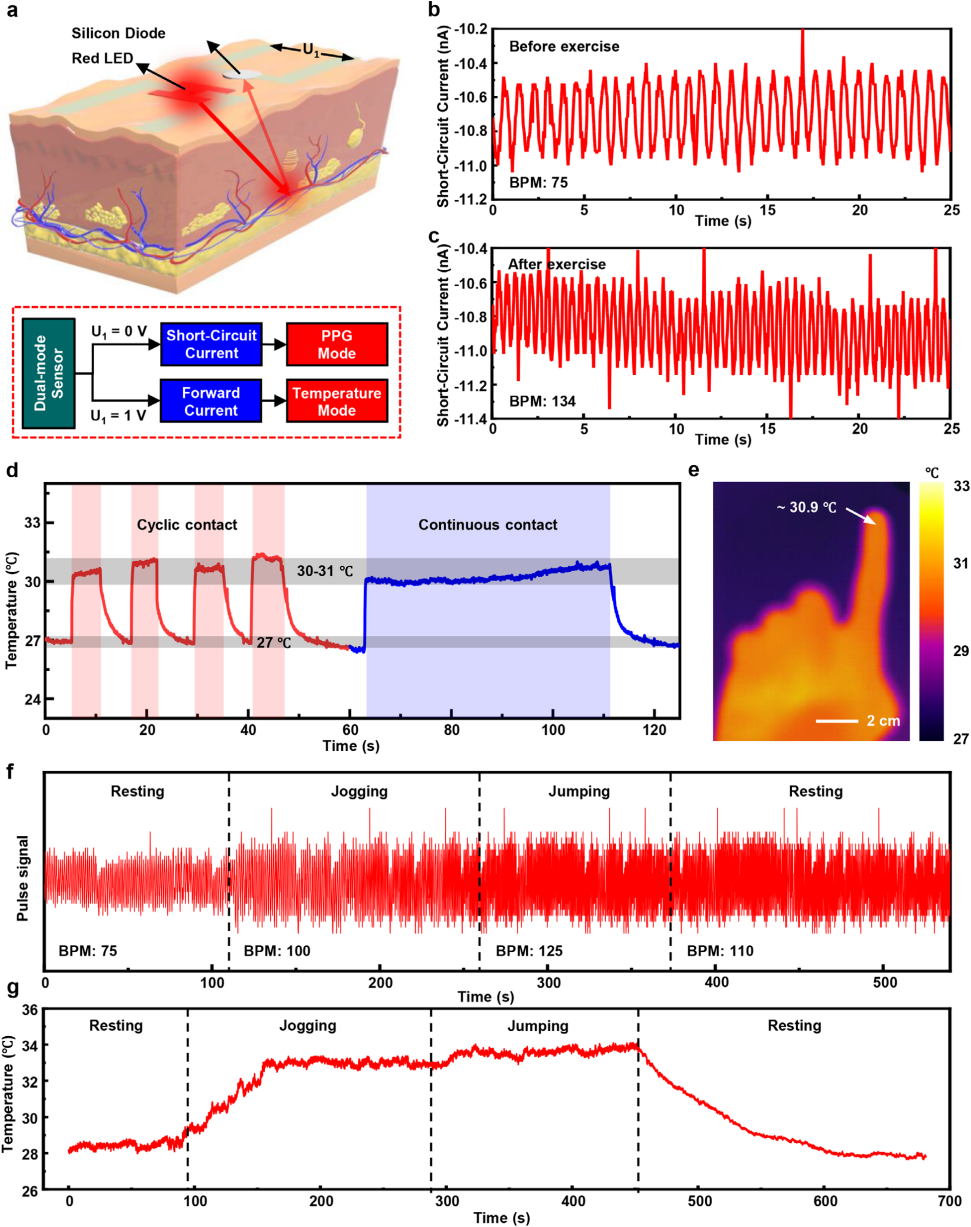
a) Schematics and operation principles of the flexible dual‐mode sensor for continuous monitoring of PPG and skin‐temperature. Continuous monitoring of the heart beating rate of an adult male before b) and after c) exercise. d) Continuous monitoring of the fingertip temperature, under cyclic (red curve) and continuous (blue curve) contacts, by the sensor operated at the temperature sensing mode. e) Infrared image of the hand revealing the corresponding temperature distribution. f) Continuous monitoring of the heart beating rate of an adult male during different exercising statuses. g) Continuous monitoring of the fingertip temperature of an adult male during different exercising statuses.

Both experimental and theoretical results reveal that the fabricated flexible Si‐based diode is capable of decoupling the crosstalk between light and temperature. As an example, a flexible dual‐modal sensing system for continuous monitoring of PPG and skin temperature is constructed, as schematically illustrated in the top panel of **Figure** **5**a. The corresponding working principle is governed by the biased condition of the Si‐based diode, as shown in the bottom panel of Figure 5a. When the biased voltage is zero, i.e., U_1_ = 0 V, and the LED is turned on, the flexible dual‐modal sensing system works in the PPG mode. The LED emits red light with a wavelength of 635 nm, at which the light has a relatively high penetration depth and low water absorption.^[^ ^55^ ^]^ Therefore, more light could be reflected and received by the diode. The I‐V characteristic of the utilized LED is shown in Figure S15 (Supporting Information). When the heart beats, periodic changes in blood vessel volume causes changes in the reflected light intensity. Therefore, the short‐circuit current can be utilized to determine the heart beating rate, which represents one of the most important applications for PPG technology.^[^ ^56^ ^]^ With the forward biased condition, e.g., U_1_ = 1 V, the skin temperature can be obtained by the forward current. Particularly, localized self‐heating induced by the continuous operation of the LED or the silicon‐based diode is negligible, as confirmed in Figure S16 (Supporting Information).

It should be noted that the distance between the LED and the Si‐based diode will strongly affect the obtained short‐circuit current. As shown in Figure S17 (Supporting Information), the short‐circuit current, as well as the signal to noise ratio during the PPG monitoring, of the sensor increases as the distance decreasing. When the distance is 0.2 cm, corresponding to 100% variation of the pre‐defined distance if set as 0.1 cm, the measured PPG can still be recognized. As a result, the distance is optimized as ≈0.1 cm, which can be precisely controlled by using the photolithography (Figure S18, Supporting Information). Figure 5b,c shows the real‐time variations of the short‐circuit current, obtained from the sensor that is attached to the fingertip of an adult male. The heart beating rate is estimated as 75 beats per minute (BPM). After doing the jogging for 20 min, the heart beating rate rises to 134 BPM. Moreover, with the forward bias of 1 V, real‐time monitoring of the skin temperature variation when the fingertip cyclically or continuously contacts with the sensor is also realized, as presented in Figure 5d. The skin temperature of the fingertip measured by the sensor is ≈30–31 °C, which is consistent with the result obtained from an infrared camera, as displayed in Figure 5e. To assess the sensing stability of the sensor, the pulse and skin temperature of an adult male are monitored during different exercising statuses, including initial, resting, jogging, jumping, and post‐resting. As shown in Figures 5f,g, during the initial resting stage, the BPM and fingertip temperature are ≈75 and 28 °C, respectively. During the jogging stage, the BPM increases to 100 and the fingertip temperature gradually rises to 33 °C. The following jumping will lead to the increase of the BPM to 125 while the fingertip temperature is relatively stable. Then, the final resting will reduce both BPM and fingertip temperature. Magnified pulse signals under different exercising statuses can be found in Figure S19 (Supporting Information). These presented results reveal that the fabricated flexible dual‐modal sensing system has good mechanical durability.

## 3 Conclusion

Concepts reported here establish a baseline of device designs, fabrication schemes, working principles, and applications for a Si‐based flexible dual‐modal sensor, which is able to realize the real‐time, independent, and continuous monitoring of both light and temperature without crosstalk. The Si‐based diodes are a critically important component of the flexible dual‐modal sensor because of their reliable and stable performances for either photodetection or temperature sensing. Moreover, both experimental characterizations and theoretical analyses demonstrate that the two operation modes (i.e., photodetection and temperature sensing) of the sensor are conveniently switchable by controlling the bias condition of Si‐based diode. The short‐circuit current (or reverse saturation current) and forward current are capable of determining light and temperature, respectively, with negligible crosstalk especially for the small variation range of the skin temperature and the low light power density for the PPG application. As an example, continuous monitoring of heart beating rate and skin temperature are demonstrated by the flexible dual‐modal sensor attached on the fingertip. Scaling such flexible dual‐modal sensor as a sensing array configuration, of which two or more types of LEDs with different wavelengths are integrated, will further expand opportunities for applications in multi‐site PPG and temperature monitoring.

## 4 Experimental Section

### Fabrication of the PI Substrate

The fabrication of PI substrate began with a glass cleaned with acetone, isopropyl alcohol, and deionized water. Spin‐coating of poly (methyl methacrylate) (PMMA, 950 PMMA A4, MicroChem), which was baked on a hot plate at 180 °C for 2 min formed a sacrificial layer. Then, poly (pyromellitic dianhydride‐co‐4,4′‐oxydianiline) amic acid (PI, Vespel^@^ SP‐21, DuPont Inc.) was spin‐coated at 1500 rpm for 30 s, followed by pre‐baking on a hot plate at 80 °C for 1 min. Fully curing at 270 °C for 30 min created the PI substrate on the top of PMMA.

### Fabrication of the Flexible Dual‐Modal Sensor

The fabrication of flexible dual‐modal sensor began with a clean silicon‐on‐insulator (SOI) wafer (see Figure S1, Supporting Information), in which the thicknesses of top silicon (P‐type, resistivity: 0.04–0.1 Ω.cm) and buried oxide layer are 2 µm and 500 nm, respectively. Then, phosphate‐based spin‐on‐dopant (P509, Filmtronics) was coated and annealed at 1000 °C for 10 min to complete doping. Photolithography and reactive ion etching through a photoresist mask (AR‐P 5350, Allresist Inc.) defined the diode with a diameter of 100 µm. After that, the transfer of patterned silicon on a flexible PI substrate was realized by the photoresist‐anchored transfer printing technique,^[^ ^57^, ^58^ ^]^ after which the residual photoresist was removed by oxygen plasma cleaner. Prior to the device fabrication, the native oxides of silicon were removed by the immersion in 30:1 buffered oxide etchant for 30 s. Then, atomic layer deposition system was used to deposit the passivation layer of Al_2_O_3_ with a thickness of 15 nm, which was patterned to expose the electrode regions by photolithography and reactive ion etching. Ti/Pt electrodes and interconnects were deposited by the sputtering, followed by the annealing treatment at 270 °C for 4 min in N_2_. Immersion in acetone for 12 h removed the PMMA sacrificial layer. Finally, the flexible dual‐modal sensor was fabricated by mounting an LED (0805, Telesky) at the Ti/Pt interconnects by Ag‐based solder pastes (BASE‐SCD1, Prtronic Inc.).

### Characterizations

To evaluate the optoelectronic performances of the sensor, a 635 nm laser (MRL‐III‐635, CNI Laser) was used as the light source, with the light power density being measured via a light power meter (LP‐3A, Beijing Material Science). An optical chopper (OE3001, SINE SCIENTIFIC INSTRUMENTS) was used to provide a pulsed light signal. For the temperature sensing test, a hot plate (LC‐MSB‐HD, LICHEN) was used to control the applied temperature. A commercial thermometer (AS877, SMART) was used for the calibration. A source meter (B2902B, Keysight Technologies) was used to measure the I–V characteristics of the sensor. An Infrared camera (D384M, Guide sensmart) was used to measure the skin temperature of fingertips.

### Human Subject Study

The experiment involving human participants was approved by the Science and Technology Ethics Committee in the School of Integrated Circuits at Shandong University (Approval No. 20 241 008). A 25‐year‐old male was recruited for the study after being fully informed about the research and providing written informed consent.

### Statistical Analysis

No data preprocessing has been employed. Data in line charts are displayed as mean ± SD. The sample size (N) was indicated in the figure captions and Experimental Section. Origin has been used for data processing.

## Conflict of Interest

The authors declare no conflict of interest.

## Supporting information



Supporting Information

## Data Availability

The data that support the findings of this study are available in the supplementary material of this article.
