# Supplementary material for: Flexible Dual‐Modal Sensors Based on Single‐Crystalline Silicon Membranes for Continuous Monitoring of Photoplethysmography and Skin Temperature
Source: Adv Sci (Weinh). 2025 Jun 25;12(35):e06348. doi: 10.1002/advs.202506348 (PMC12463106; doi:10.1002/advs.202506348)
Supplement: Supplementary file 1 — Supporting Information [file ADVS-12-e06348-s001.pdf]

ADVANCED SCIENCE
Open Access

Supporting Information

for *Adv. Sci.*, DOI 10.1002/advs.202506348

Flexible Dual-Modal Sensors Based on Single-Crystalline Silicon Membranes for Continuous Monitoring of Photoplethysmography and Skin Temperature

*Yanle He, Haonan Zhao, Xiaozhong Wu, Junjie Zhou, Ailing Yin, Deyu Meng, Wenli Sun and Qinglei Guo**

Supporting Information

Flexible Dual-Modal Sensor Based on Single-Crystalline Silicon Membranes for Continuous Monitoring of Photoplethysmography and Skin Temperature

*Yanle He, Haonan Zhao, Xiaozhong Wu, Junjie Zhou, Ailing Yin, Deyu Meng, Wenli Sun, and Qinglei Guo**

Supplementary Note 1 to Figure S13:

To identify the operational limit of temperature, variations in the short-circuit current of sensor, which is illuminated with a constant light power density (0.5 mW/mm^2), with the temperature are extracted. The reason for setting the light power density as 0.5 mW/mm^2 is that most of commercially available PPG sensors use light emitting diodes (LEDs) with light power densities less than 0.5 mW/mm^2, as shown in Table S3. As shown in Figure S13a, the short-circuit current of the sensor is almost stable at the low temperature range, and followed by significant increases once the temperature exceeds a certain value. The right axis of Figure S13a represents the calculated pseudo light power density by utilizing the short-circuit current, also referring to the results in Figure 2b. Notably, the temperature at which the relative change between the calculated pseudo light power density and the actual one (i.e., 0.5 mW/mm^2) reaches 1% is intentionally defined as the operational limit. As a result, the operational limit of temperature is approximately 60 ℃, which is higher than the normal temperature of human.

For the operational limit of light power density, variations in the forward current of sensor at 37 ℃ as the light power density rises are assessed. The reason for setting the temperature as 37 ℃ is that it represents the average temperature for healthy human body. As shown in Figure S13b, the forward current decrease as the increase of the light power density. The right axis represents the calculated pseudo temperature by utilizing the forward current, also referring to the results in Figure 2d. Notably, the light power density at which the change between the calculated pseudo temperature and the actual one (i.e., 37 ℃) reaches 0.1 K is intentionally defined as the operational limit. As a result, the operational limit of light power density is approximately 33 mW/mm^2, which is higher than that reqiured for commercial PPG sensors as shown in Table S3.

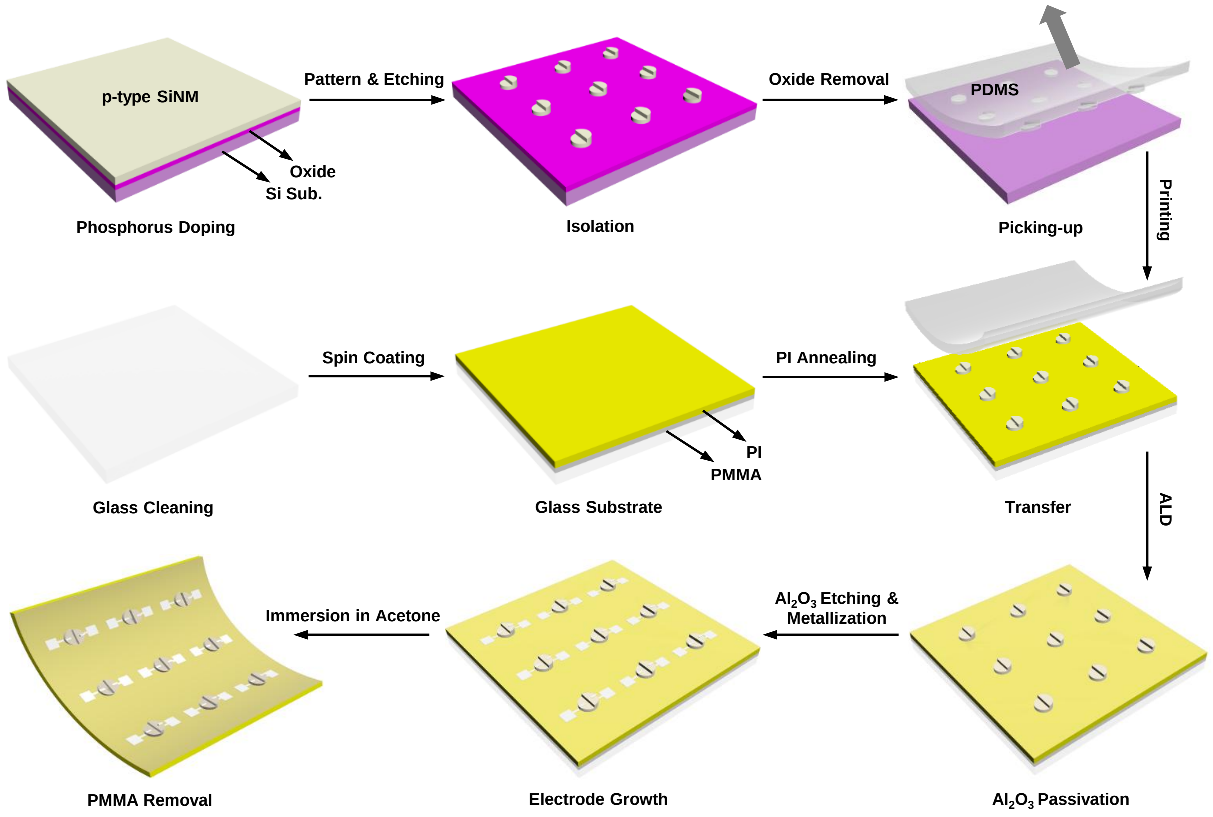

Figure S1. Schematic illustration of key fabrication steps of flexible silicon-based diodes.

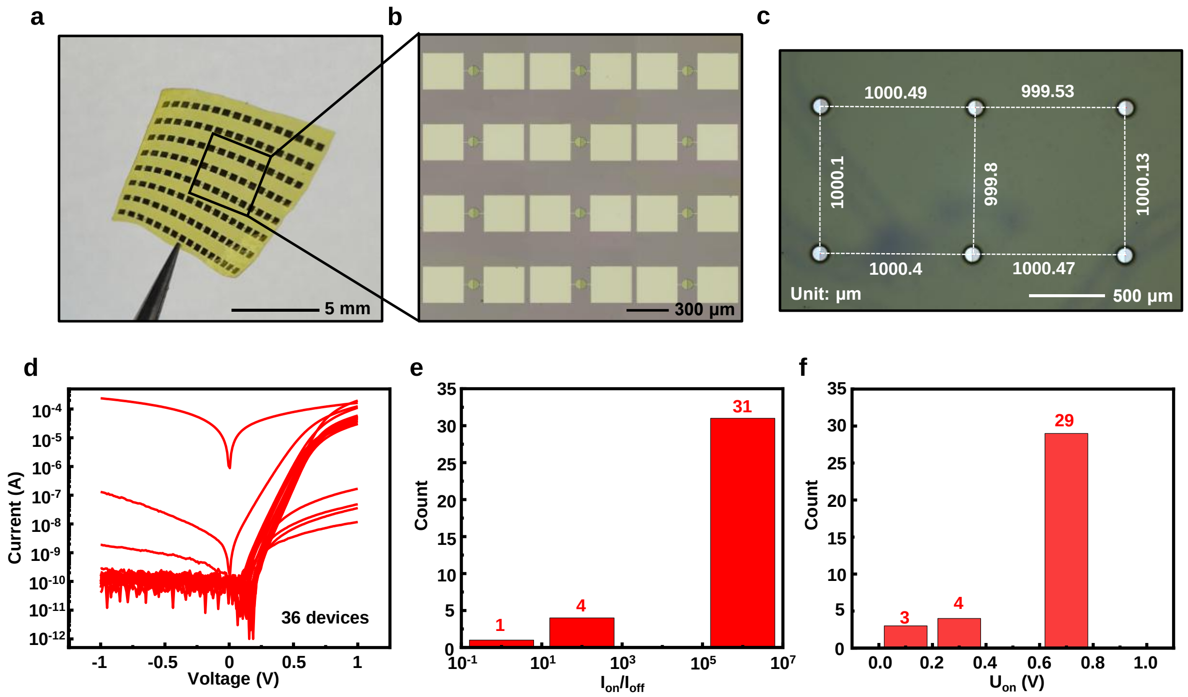

Figure S2. (a) Optical image of a 6×6 flexible silicon-based diode array. (b) Magnified image of the device array captured by an optical microscopy. (c) Optical microscope image of the patterned silicon nanomembrane array transferred to flexible PI substrate. (d) Current-voltage characteristics of 36 silicon diodes from the fabricated 6×6 flexible diode array. Statistical results of the I_{on}/I_{off} (e) and the turn-on voltage (f) extracted from the data in Figure S2d.

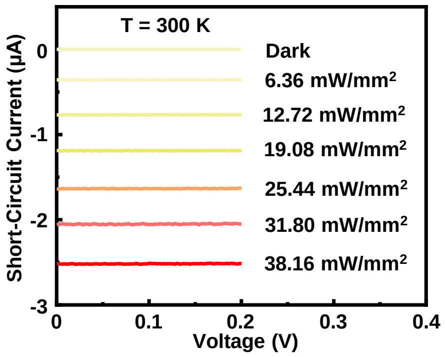

Figure S3. Magnified current-voltage characteristics of the fabricated flexible silicon-based diode under illumination with different light power densities.

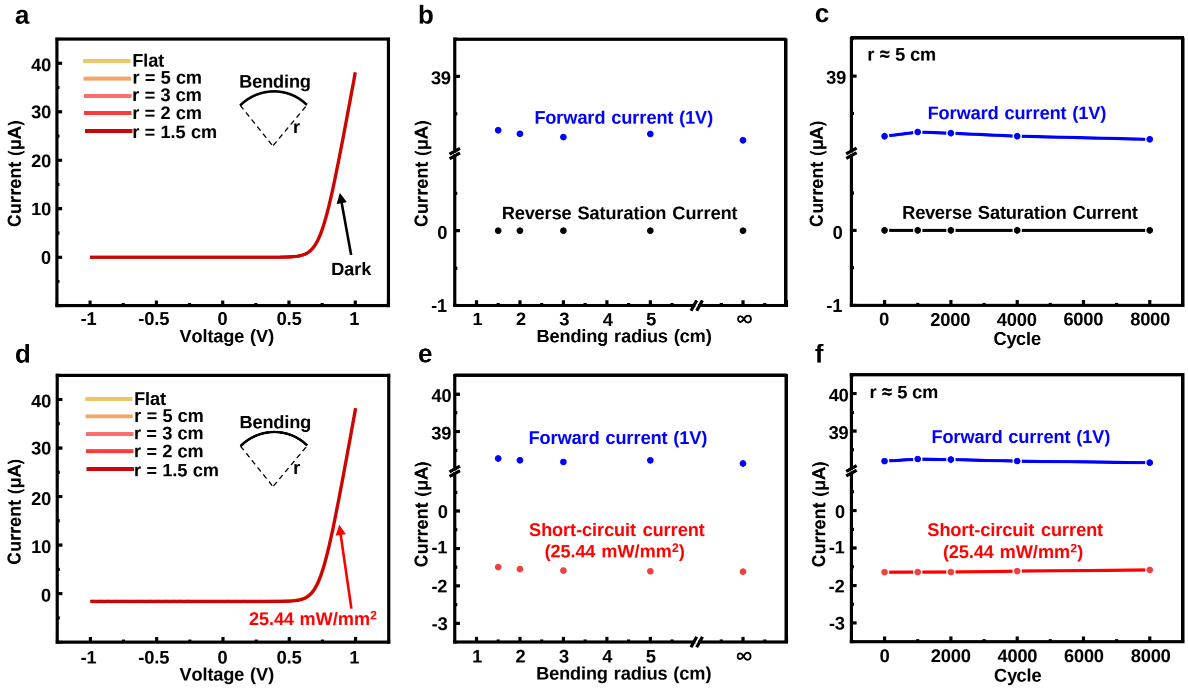

Figure S4. Current-voltage characteristics of silicon-based diode under different bending radii without (a) and with (d) light illumination. Variations of the reverse saturation current and the forward current (bias voltage: 1 V) of the sensor under dark condition with the bending raduis (b) and the bending cycles (c). Variations of the reverse saturation current and the forward current (bias voltage: 1 V) of the sensor under light illumination condition with the bending raduis (e) and the bending cycles (f). During the cycled bending tests, the bending radius (r) is 5 cm.

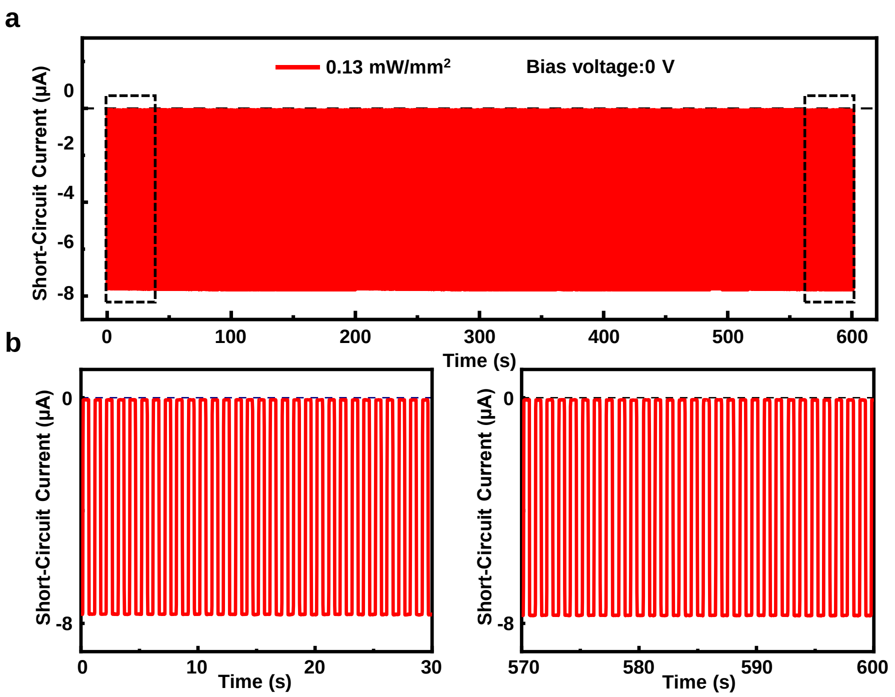

Figure S5. (a) Short-circuit current variations of the sensor illuminated by a pulsed laser with the frequency and power density of 1 Hz and 0.13 mW/mm^2, respectively. (b) Magnified results of the short-circuit current variations highlighted with two dotted, black rectangles in (a).

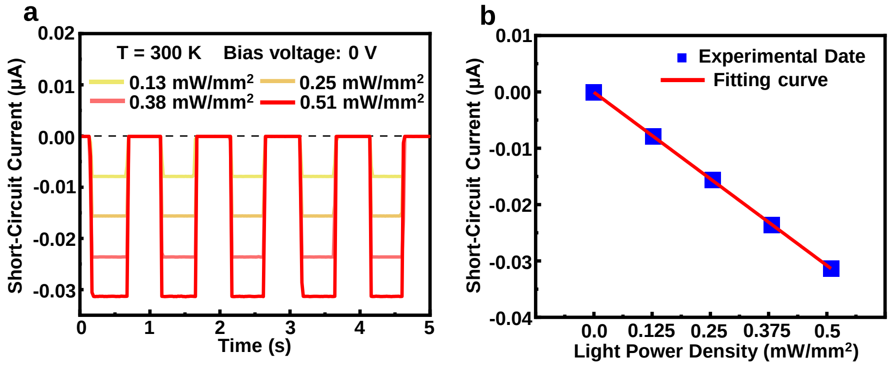

Figure S6. (a) Short-circuit current variations of the sensor illuminated by a pulsed laser with different light power densities at 300 K. (b) Experimental (blue dots) and fitting (red line) results of the variations in short-circuit current under different light power densities.

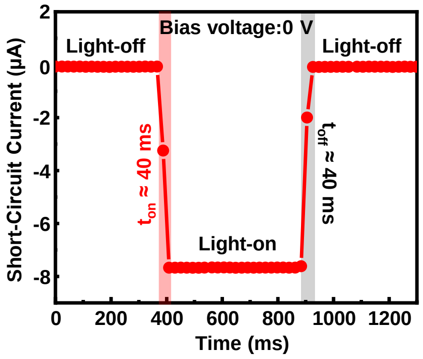

Figure S7. The response time of the sensor extracted from one cycle of light illumination.

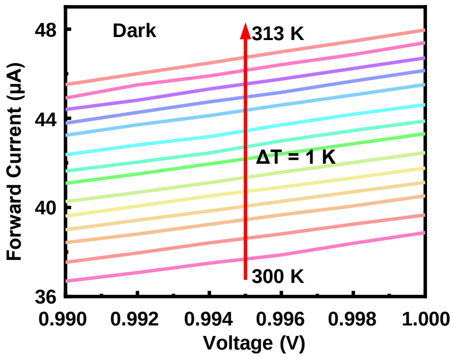

Figure S8. Current-voltage characteristics of the fabricated flexible silicon-based diode at different temperatures.

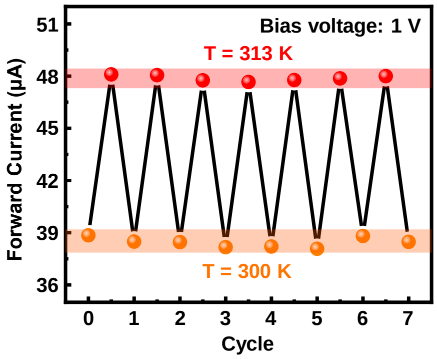

Figure S9. Forward current variations during cycled heating (313 K) and cooling (300 K) treatments of the sensor biased at 1 V.

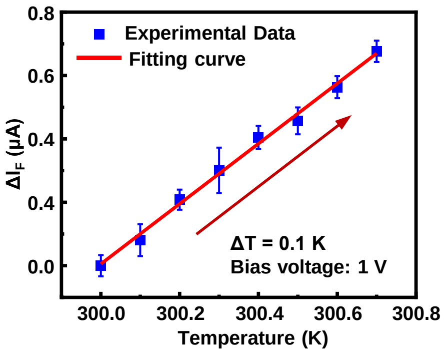

Figure S10. Forward current variations of the sensor at different temperatures with a step-rising of 0.1 K. Data shown as mean ± standard deviation. All experiments were repeated at least 5 times using independent samples (N = 5).

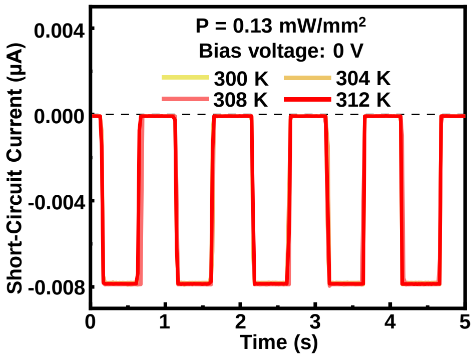

Figure S11. Short-circuit current variations of the fabricated flexible silicon-based diode that is illuminated with a constant light power density, and the temperature varies from 300 K to 312 K with an interval of 4 K.

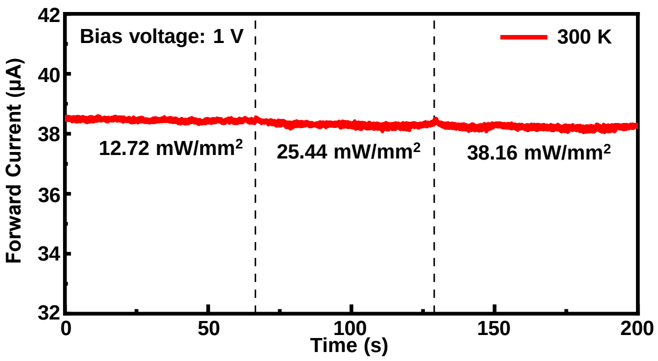

Figure S12. Forward current variations of the fabricated flexible silicon-based diode at a constant temperature with varies light power densities.

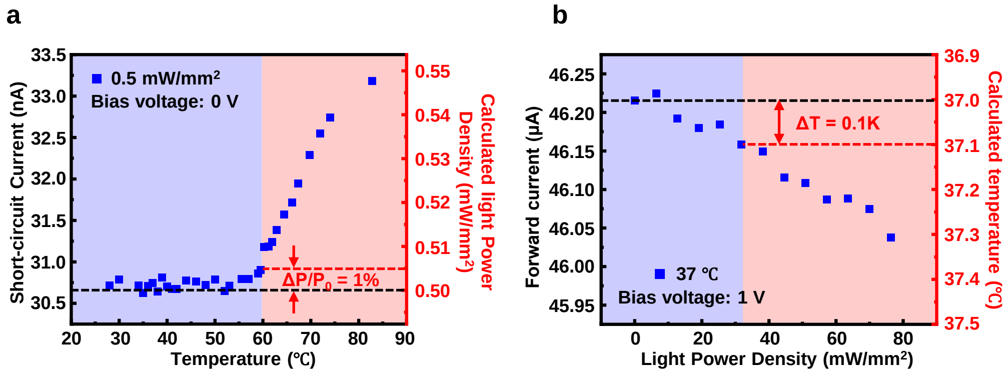

Figure S13. (a) Short-circuit current of the sensor, which is illuminated by a 635 nm laser with a power density of 0.5 mW/mm^2 varying with the temperature. The right axis represents the calculated pseudo light power density. (b) Forward current of the sensor at 37℃ varying with the light power density. The right axis represents the calculated pseudo temperature.

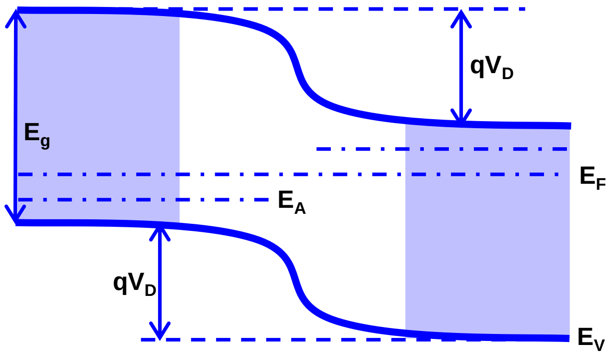

Figure S14. Energy band diagram of silicon-based PN junction under equilibrium condition.

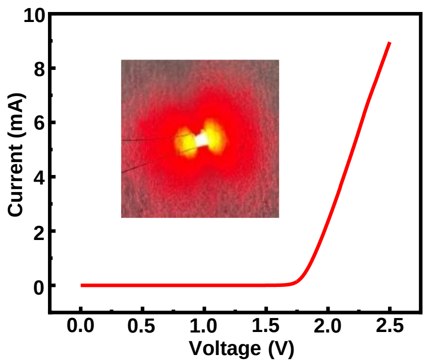

Figure S15. Current-voltage characteristics of the utilized red LED. The inset is an optical image of the LED that is turned-on.

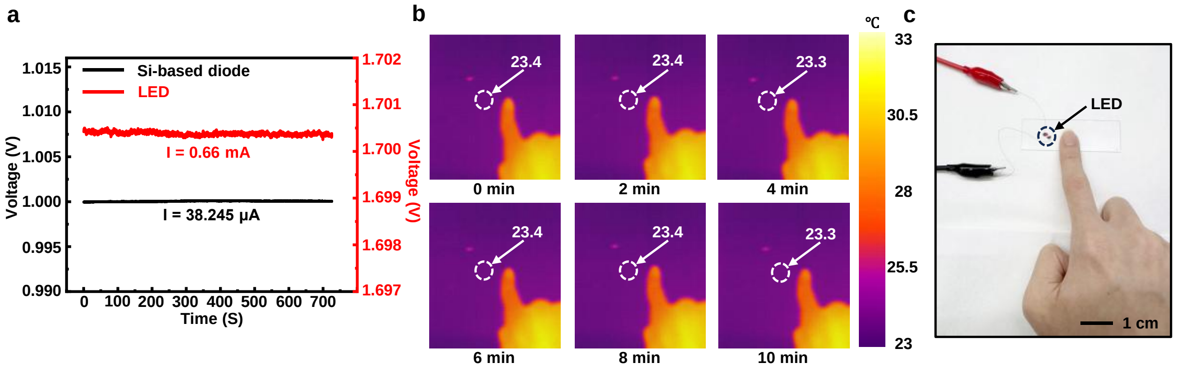

Figure S16. (a) Long-term variations in the output voltage of the silicon-based diode (black) and the LED (red) with continuous input of a constant current of 38.245 μA and 0.66 mA, respectively. (b) Infrared images of the LED at different operation periods. (c) Optical image of the LED in operation.

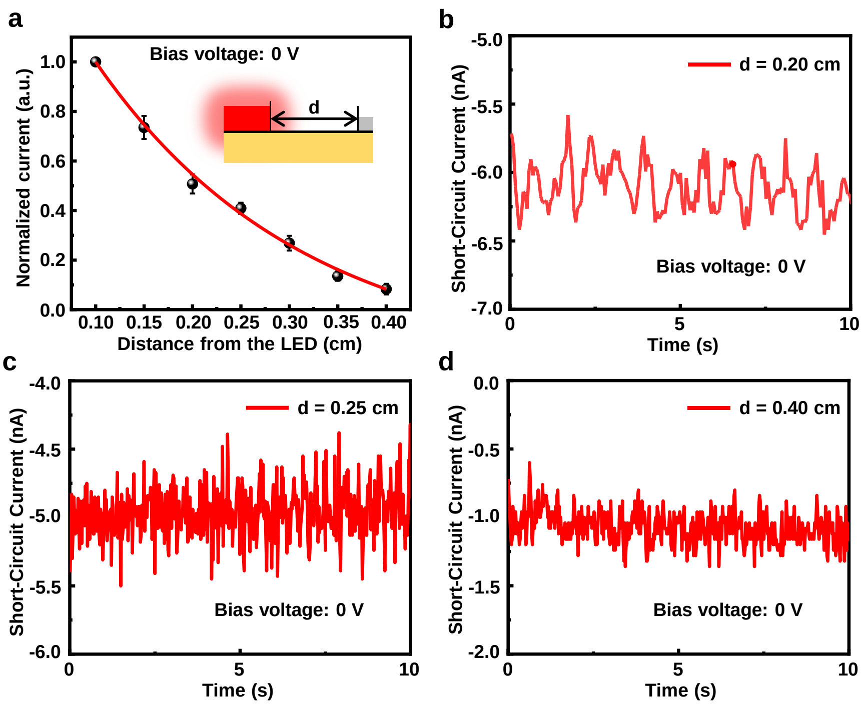

Figure S17. Optimization of distance between red LED and silicon-based PN junction. (a) Normalized short-circuit current obtained from the silicon-based diode varying with the distance from the LED. Measured PPG signals by the sensor with a distance between silicon-based diode and LED of 0.2 cm (b) ,0.25 cm (c) and 0.4 cm (d). Data shown as mean ± standard deviation. All experiments were repeated at least 3 times using independent samples (N = 3).

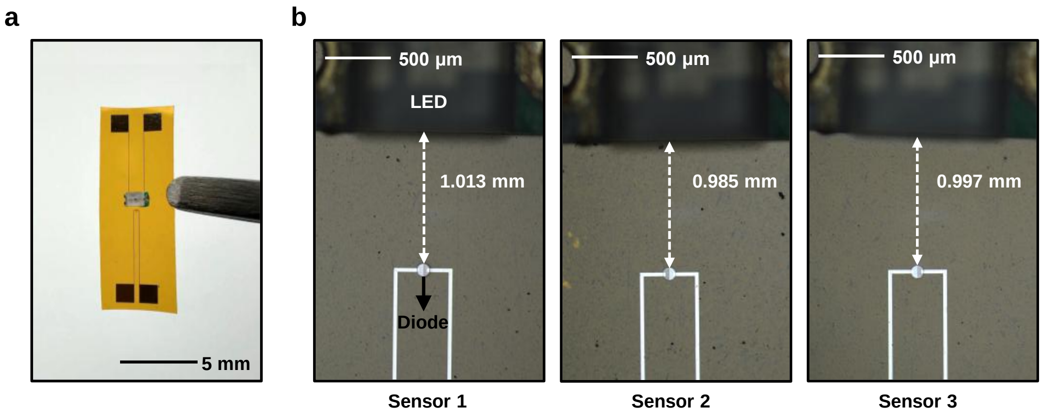

Figure S18. (a) Optical image of a typical flexible dual-mode sensor. (b) Optical microscope images of three fabricated devices. The average of distance between diode and LED is 0.998±0.015 mm.

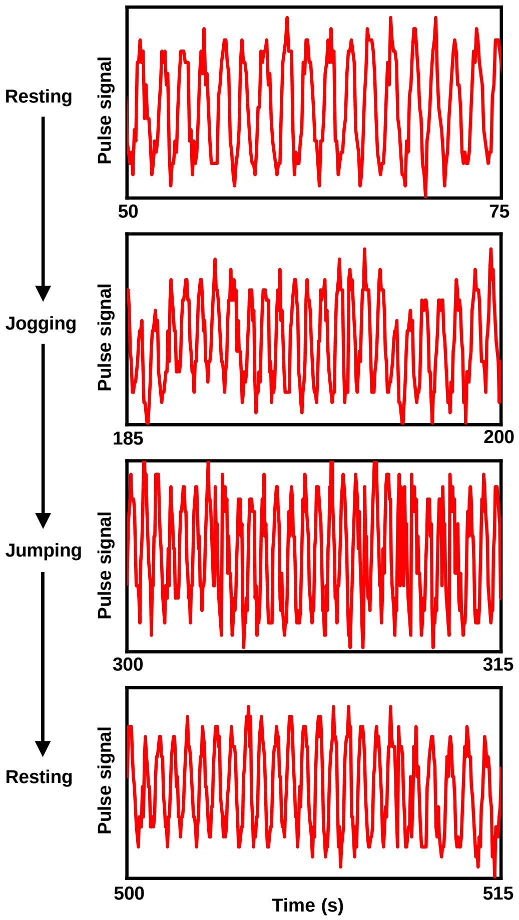

Figure S19. Magnified results of the pulse signal at different conditions in Figure 5f.

Table S1. Comparisons on the temperature coefficient of resistance (TCR) or current (TCC) of the fabricated SiNM-based temperature sensors with previously reported works.

Ref	Materials	Device	|TCR| or |TCC|
[1]	Au-doped SiNM	Resistor	3.727 %/ ^{o}C
	B-doped SiNM	Resistor	0.169 %/ ^{o}C
[2]	B-doped SiNM	Resistor	0.1 %/ ^{o}C
[3]	P-doped SiNM	Resistor	0.12 %/ ^{o}C
[4]	B-doped SiNM	Resistor	0.1 %/ ^{o}C
[5]	P-doped SiNM	Resistor	0.23 %/ ^{o}C
This work	SiNM-based PN junction	Diode	1.79 %/ ^{o}C

Table S2. Comparisons on the key parameters of the dual-modal sensors with previously reported works.

Ref	Materials	Sensing Function	Sensitivity	Resolution	Size
[6]	Nb-doped $SrTiO_3$	Light<br>Temperature	0.201 μA/(mW·cm^{-2}) (980 nm)<br>16.77 μA/K	-<br>0.5 K	100 mm^2
[7]	Ce-doped HfO_2	Light<br>Temperature	Nonlinearity (808 nm)<br>894.7 μC·m^{-2}·K^{-1}	-<br>0.1 K	1 mm^2
[8]	Au/N-BuHHPDI*/Au	Humidity<br>Light<br>Temperature	Nonlinearity<br>Nonlinearity (sun)<br>Nonlinearity	-<br>-<br>-	-
[9]	ITO/ZnO/ITO	Light<br>Temperature	30.69 μA/(W·cm^{-2}) (365 nm)<br>4.89 μA/K	-<br>-	0.85 cm^2
[10]	SnO_2/MoS_2/RGO*	Humidity<br>Light<br>Temperature<br>CO_2	2 MHz/%RH<br>0.02 dB/Lux (sun)<br>0.63 dB/K<br>0.012 dB/Lux	-<br>-<br>-<br>-	846 mm^2
[11]	DTe-WPU*	Light<br>Temperature	-<br>0.165 μA/K#	-<br>-	-
This work	Si	Light<br>Temperature	0.07 μA/(mW·mm^{-2}) (635 nm)<br>0.65 μA/K	-<br>0.1 K	0.007 mm^2

*Full names of abbreviations are: BuHHPDI: N-Butyl-N′-(6-hydroxyhexyl) perylene-3,4,9,10-tetracarboxylic acid diimide. RGO: Reduced Graphene Oxide. DTe-WPU: multifunctional waterborne polyurethane chemical crosslinked network. #Calculated values based on the data in the reference.

Table S3. Light power density of LED in various commercial PPG sensors.

Manufacturer	Product model	LED type	Range of light power density (mW/mm^2)
Omron	HPO-200T	Green, IR	0.25-0.4
Analog Device	MAX30102	Green, IR	0.2-0.5
AMS	AS7341	Red	0.3-0.5
Senbiosys	SB140M1	Green, Red, IR	0.2-0.5
Panasonic	PAN1710	Green, Red, IR	0.15-0.4
Yuwell	YX106	Red, IR	0.4-0.5
Latticepower	-	Green, Red, IR	0.3-0.5

References:

1. M. Sang, K. Kang, Y. Zhang, H. Zhang, K. Kim, M. Cho, J. Shin, J. H. Hong, T. Kim, S. K. Lee, W. H. Yeo, J. W. Lee, T. Lee, B. Xu, K. J. Yu, Advanced Materials **2021**, *34*, 2105865.
2. K. Kwon, J. U. Kim, S. M. Won, J. Zhao, R. Avila, H. Wang, K. S. Chun, H. Jang, K. H. Lee, J.-H. Kim, S. Yoo, Y. J. Kang, J. Kim, J. Lim, Y. Park, W. Lu, T.-i. Kim, A. Banks, Y. Huang, J. A. Rogers, Nature Biomedical Engineering **2023**, *7*, 1215.
3. J. Shin, Y. Yan, W. Bai, Y. Xue, P. Gamble, L. Tian, I. Kandela, C. R. Haney, W. Spees, Y. Lee, M. Choi, J. Ko, H. Ryu, J.-K. Chang, M. Pezhouh, S.-K. Kang, S. M. Won, K. J. Yu, J. Zhao, Y. K. Lee, M. R. MacEwan, S.-K. Song, Y. Huang, W. Z. Ray, J. A. Rogers, Nature Biomedical Engineering **2018**, *3*, 37.
4. S.-K. Kang, R. K. J. Murphy, S.-W. Hwang, S. M. Lee, D. V. Harburg, N. A. Krueger, J. Shin, P. Gamble, H. Cheng, S. Yu, Z. Liu, J. G. McCall, M. Stephen, H. Ying, J. Kim, G. Park, R. C. Webb, C. H. Lee, S. Chung, D. S. Wie, A. D. Gujar, B. Vemulapalli, A. H. Kim, K.-M. Lee, J. Cheng, Y. Huang, S. H. Lee, P. V. Braun, W. Z. Ray, J. A. Rogers, Nature **2016**, *530*, 71.
5. D. Meng, H. Zhao, X. Wu, M. Liu, Q. Guo, IEEE Electron Device Letters **2024**, *45*, 2518.
6. L. Wu, Y. Ji, B. Ouyang, Z. Li, Y. Yang, Advanced Functional Materials **2021**, *31*, 2010439.
7. J. Peng, J. Jiang, S. Yuan, P. Hou, J. Wang, Journal of Materiomics **2025**, *11*, 100911.
8. M. Tahir, M. H. Sayyad, J. Clark, F. Wahab, F. Aziz, M. Shahid, M. A. Munawar, J. A. Chaudry, Sensors and Actuators B: Chemical **2014**, *192*, 565.
9. T. Gao, Y. Ji, Y. Yang, *Advanced Materials Technologies* **2020**, *5*, 2000176.
10. F. Miao, Y. Han, P. Tian, B. Tao, Y. Zang, P. K. Chu, *Sensors and Actuators B: Chemical* **2023**, *390*, 133913.
11. W. Zeng, Y. Jin, R. Zhou, Y. Li, H. Chen, *Chemical Engineering Journal* **2024**, *482*, 184994.
